# A new approach to identifying safety measures across transfers of care for people who use insulin for Type 2 diabetes

**DOI:** 10.1111/dme.70101

**Published:** 2025-07-22

**Authors:** Catherine Leon, Clare Crowley, Helen Hogan, Yogini H. Jani

**Affiliations:** ^1^ Department of Health Services Research and Policy London School of Hygiene & Tropical Medicine London UK; ^2^ Reading School of Pharmacy University of Reading Reading UK; ^3^ Department of Practice and Policy University College London School of Pharmacy London UK; ^4^ Centre for Medicines Optimisation Research and Education University College London Hospitals NHS Foundation Trust London UK

**Keywords:** complications, Diabetes, drug safety, insulin

## Abstract

**Aims:**

When people who use insulin for Type 2 diabetes have a hospital admission and discharge, they are at risk of harm from incorrect, delayed, or missed insulin doses. Leading indicators can highlight potential areas of risk, providing opportunities to improve safety. Modelling the complex transfer of care pathway can provide insight into where leading indicators could be targeted to support improved outcomes.

**Methods:**

Multiple qualitative methods were used, and a framework approach was applied to identify activities (termed functions) involved in managing insulin during the transfer of care, and how factors involving people, equipment and environments (local, organisational and external) impacted these. The Functional Resilience Analysis Method was used to map the transfer of care pathway, and key areas of variability were identified. These areas of variability and two example functions were validated and discussed with key/representative stakeholders in an online seminar.

**Results:**

A total of 59 functions were mapped, and 21 were identified as key functions for potential new measures. These 21 functions were validated at a seminar, and two example functions, empowering people with diabetes who use insulin to manage their diabetes and arranging self‐administration of insulin in hospital, were discussed in detail. A selection of potential measures was identified.

**Conclusions:**

Many potential areas for new leading indicators were identified, and examples of potential measures were described. A coproduction approach is required to expand, define and validate these. Such measures provide an opportunity for proactively improving insulin safety during care transfers.

**What's new?**

**What is already known?**

- Transfers of care for people with diabetes are known to be challenging for safe insulin management.

**What has this study found?**

- The Functional Resonance Analysis Method (FRAM) was used to identify areas of variability as potential targets for proactive indicators of safe insulin management during transfer of care.

**What are the implications of this study?**

- FRAM is a successfully applied novel approach for identifying potential leading indicators and provides new areas for testing and validation.

## 1 BACKGROUND

People with diabetes who use insulin (PWDI) face risks when they have a hospital admission and then return home.^1^, ^2^, ^3^, ^4^ Inadvertent changes to insulin prescriptions occur frequently and can cause significant harm to PWDI.^5^, ^6^, ^7^, ^8^ The process of moving between care settings, such as home and hospital, is called Transfer of Care (ToC). To manage insulin safely and avoid harm, it is important to communicate how insulin should be given and adjusted, and to consider the effects of illness, changes in diet and activity levels, and other medications. If insulin doses are incorrect, missed, or delayed, it can cause serious harm or even death.

Over the years, patient safety campaigns have tried to improve insulin management during ToC by creating patient‐held insulin records,^4^ developing guidelines for self‐administration,^9^ and introducing e‐learning for patients and staff.^10^, ^11^ Despite these efforts, the same issues remain prevalent and continue to be addressed in a national campaign in England called the Get it Right First Time (GIRFT) Diabetes program.^12^

To improve insulin safety during ToC, it is essential to have measures that demonstrate how well insulin is being managed. Without this data, it is hard to prove the need for change, identify where to focus improvements, or see if interventions are working. Most traditional measures look at harm rates, like rates of hypo‐ and hyperglycaemia, hospital readmissions, and deaths.^13^ With increased digitisation of health records across health and care sectors, there is opportunity and a need for predictive measures, called leading indicators, that can identify risks in real time and allow proactive safety improvements. These measures could help healthcare teams, organisations, and policymakers develop better systems for safe insulin management.

Healthcare is a complex system made up of people, tasks, equipment, and different environments.^14^ These factors evolve, interact and change, requiring adjustments in care.^15^ To measure safety effectively, it is important to understand these interactions and adjustments and how they combine to create variation in outcomes.^16^, ^17^

Leading indicators proactively highlight areas that may need to be addressed to prevent or minimise harm. There are two types of leading indicators, active and passive.^18^ Active leading indicators for use by people directly involved in providing and receiving care, such as the National Early Warning Sign scores, can highlight people at risk of deterioration in real time, prompting timely review.^19^ Passive leading indicators provide information to organisations about how well the systems and processes are designed.^18^

The Functional Resonance Analysis Method (FRAM) is a tool that models complex systems and examines how aspects of variability affect outcomes.^20^ FRAM has been used to develop indicators for detecting sepsis.^17^ FRAM models can represent both daily activities (termed functions) and the structural factors that support them (background functions). This study aimed to use FRAM to identify where variation due to interacting system factors can impact outcomes for safe insulin management during ToC. These areas were considered as targets for developing proactive indicators to highlight, in real time, opportunities for safety interventions. Such indicators will complement traditional indicators in improving safety for this patient group.

## 2 METHODS

### 2.1 Study design

**FIGURE 1:**
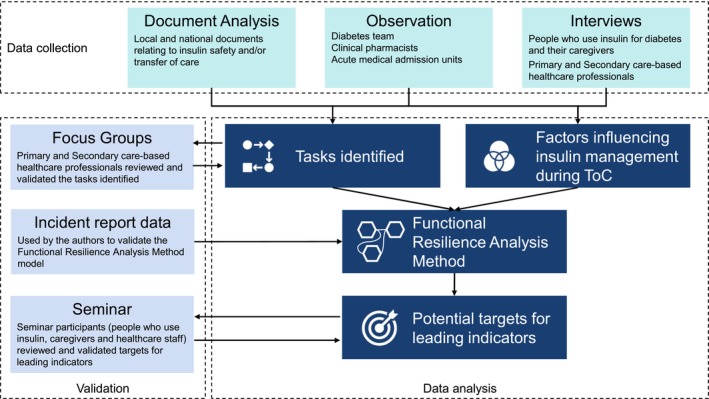
Data sources and components of research.

Multiple qualitative methods were used to develop an understanding of insulin management for people with Type 2 diabetes during ToC. An overview of the components of the study is shown in Figure 1. ToC was defined as being from when the need for hospital admission was identified through to routine follow‐up after discharge.

### 2.2 Setting and sample

Fieldwork was undertaken over 17 months between October 2022 and March 2024. The setting was initially within an integrated care system in England. To boost numbers, recruitment was widened to include subjects for interviews, focus groups, and the seminar from across England. Full details of all data sources (documents, observations undertaken and interview, subjects) are included in the Data S1.

### 2.3 Data collection

Documentary analysis was undertaken on national and local documents relating to insulin safety and/or transfer of care.

Purposive observation was undertaken over 85 h in a large, acute teaching hospital. Field notes were written during and immediately following the period of observation. The Systems Engineering Initiative for Patient Safety 101 (SEIPS) work system categories were used to guide observations.^21^ These categories define a work system as composed of people, tools and equipment, tasks and environments (local, organisational and external).

Semi‐structured online interviews were undertaken with people involved in managing insulin during ToC, including PWDI over 18 years with Type 2 diabetes and a hospital admission within the last 5 years, or their caregivers and multiple professions across primary and secondary care. PWDI or caregivers were excluded if they could not participate in a telephone call or online video call or required an interpreter. Twenty interviewees were asked to describe their experiences with managing insulin during ToC, what goes well and where challenges are involved. Subjects were identified by healthcare professionals during observation and through invitations shared on national diabetes forums and on social media. Purposive sampling of healthcare professionals known to the authors was used to invite participation in the interviews and online seminar.

### 2.4 Data analysis

Tasks required to manage insulin during ToC and the factors that influenced them were identified through a framework analysis of documents, field notes from observations and transcripts from interviews. SEIPS 101^21^ work system categories were used to guide analysis. Factors that impacted insulin management were categorised according to whether they involved tasks, people, tools or environments (local, organisational or external). FRAM is a method that supports the identification of areas of variability which have the potential to be developed as leading indicators. To best illustrate this application of this method, this paper focuses on the emergency admissions to hospital element of the ToC pathway, as this is a particularly high‐risk time for safety.

The tasks identified during framework analysis were used as the basis for the FRAM functions, according to the method defined by Hollnagel,^20^ and potential targets for leading indicators were identified using the method defined by Raben et al.^17^ The functions were evaluated according to six aspects:

1. **Input**–the prompt for the function to begin.
2. **Output**–the outcome of the function.
3. **Pre‐conditions**—anything that must be in place for the function to begin.
4. **Resources**—resources needed for the function, could be skills, equipment and guidelines for example.
5. **Controls**—the aspects of the system that control the output of the function, for example, IT programming or regulations.
6. **Time**—how time influences how the function is performed, for example, whether it needs to be before other functions, or how long the function may take to process.

**FIGURE 2:**
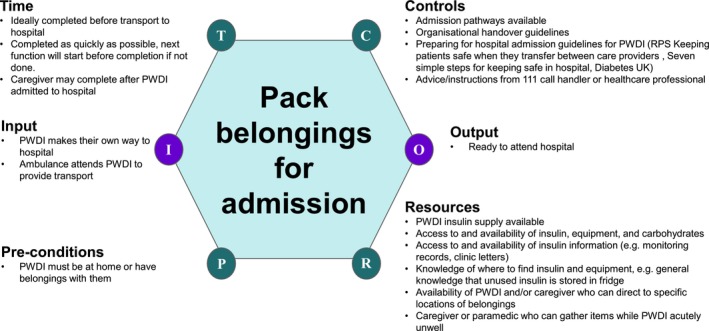
An example function demonstrating the aspects identified.

See Figure 2 for an example function.

The model was built iteratively. Further functions were identified, including background functions. Background functions impact the success of other functions (foreground functions) but are not activities within the care pathway being studied. They include factors such as appropriately trained staff and organisational policies.

Each function was reviewed to explore how it varied and how this variation might impact the outcomes for insulin management during ToC. Variation could be due to the accuracy of the function, or the timing. For example, the function could be incomplete or incorrect, performed too early, too late or omitted. The potential variability and the consequences of this variability on other functions and ToC outcomes were then described and recorded in a spreadsheet. Table S1 presents a sample of functions and their identified variability. Those functions where variability had the potential to impact outcomes were considered as potential targets for leading indicators.

### 2.5 Validation

Two focus groups were held with four healthcare professionals from primary and secondary care to agree on the completeness and accuracy of the tasks involved in managing insulin safely during ToC identified through documentary analysis, observation and qualitative interviews.

Once developed, the FRAM model was tested for completeness using incident reports from the National Reporting and Learning System (NRLS). A structured search was performed of the NRLS database to identify incidents relating to insulin and related terms, admission, discharge, and transfer of care. A random sample of 100 incident reports was accessed, covering both primary and secondary care. From these reports, 10 incidents that provided the most comprehensive narratives and represented different sections of the patient journey were selected and used to check the completeness of the model (a summary of these incidents is included in the Data S2). Suggestions for missing functions or factors and comments were requested and discussed. Additional functions identified during validation processes were added to the model as functions.

### 2.6 Identification of potential areas for safety indicator development

An online seminar was held with PWDI, caregivers and primary and secondary health professionals and managers to present the findings of the analysis and to gauge consensus on the key background and foreground functions associated with safe insulin management during ToC. Twelve PWDI, caregivers and health professionals interrogated two representative functions where variability impacted outcomes. The potential for these functions as areas for the development of new safety leading indicators was explored. The chosen functions represented one background function (empower PWDI to manage diabetes) related to the structural factors required for successful outcomes and one foreground function (arrange self management of diabetes while in hospital) related to supporting PWDI as care is being provided. The seminar identified the limits of current safety measures and measurement gaps before focusing on potential new measures related to the two functions and data collection requirements.

#### 2.6.1 Reflexivity and author contributions

Data collection and analysis was performed by the lead researcher (CL), a medication safety pharmacist by background. This allowed the author to understand the clinical context of the terms and aspects of care being observed and described. The qualitative findings were reviewed on a regular basis by the study team (YJ and HH with backgrounds in safety, pharmacy, and primary care) following which areas for further exploration and data collection were identified.

The FRAM was performed by CL with advice and feedback given by CC, a pharmacist and a Chartered Ergonomist experienced in using FRAM. Any identified need for additional functions and any differences of opinion about the model were discussed to reach consensus, and the model was updated.

### 2.7 Ethics

Ethics approval was obtained from the United Kingdom NHS Health Research Authority and Ethics Committee (22/EE/0155) and the University Ethics Committee (28148). Amendments were obtained from both ethics committees to widen the recruitment of patients, healthcare professionals, and to extend the deadline. All subjects provided informed consent for participation.

## 3 RESULTS

Documentary analysis was used to explore how work was prescribed while observation and interviews provided insight into how work was performed in an everyday setting. This allowed a detailed understanding of the ToC care pathways and the factors that influence how they are performed. This information was used to identify and define the FRAM functions, and consider where variation in function output impacts outcomes for insulin management during ToC.

Fifty‐nine functions were identified spanning ToC pathways, including nine background functions and 50 foreground functions. A list of the functions identified is included in Table 1. Analysis of incident data led to the inclusion of an additional two functions, ‘Provide authority to administer insulin for district nurses’, and ‘Review referral in primary care’.

**TABLE 1:** Functions identified for the Functional Resilience Analysis Method, with proposed key functions impacting outcomes highlighted.

	Name of function	Type of function	Proposed target for leading indicators?
1	Decide hospital admission is needed	Foreground	No
2	Pack belongings for hospital admission	Foreground	No
3	Travel to hospital	Foreground	No
4	Arrange ambulance	Foreground	No
5	Refer the person with diabetes who uses insulin (PWDI) to hospital	Foreground	No
6	Handover diabetes care to hospital	Foreground	Yes
7	Gather insulin information	Foreground	Yes
8	Monitor blood glucose levels	Foreground	No
9	Admit PWDI to hospital	Foreground	No
10	Provide orientation to clinical area	Foreground	No
11	Hospital‐based clinical team accept patient	Foreground	No
12	Confirm diabetes history	Foreground	Yes
13	Develop diabetes inpatient treatment plan	Foreground	Yes
14	Prescribe insulin	Foreground	Yes
15	Check baseline observations	Foreground	No
16	Assess blood glucose levels	Foreground	No
17	Treat hypoglycaemia	Foreground	No
18	Treat hyperglycaemia	Foreground	No
19	Arrange self‐management of diabetes for PWDI while in hospital	Foreground	Yes
20	Source insulin(s) for inpatient use	Foreground	No
21	Refer to inpatient diabetes team	Foreground	No
22	Assess and treat high ketone levels	Foreground	No
23	Adjust insulin during acute illness	Foreground	No
24	Administer routine insulin	Foreground	No
25	Perform discharge assessment	Foreground	Yes
26	Identify insulin needs for discharge	Foreground	No
27	Create insulin plan for discharge	Foreground	Yes
28	Identify equipment needs for discharge	Foreground	No
29	Arrange discharge supply of insulin & equipment	Foreground	No
30	Provide discharge letter	Foreground	Yes
31	Discharge to primary care	Foreground	No
32	Provide education to PWDI or carer	Foreground	Yes
33	Make primary care referrals	Foreground	Yes
34	Secondary care diabetes team make follow‐up phone call	Foreground	No
35	Travel home	Foreground	No
36	Manage diabetes at home	Foreground	Yes
37	Primary care team accept referral	Foreground	No
38	Identify hospital discharge	Foreground	No
39	Reconcile insulin in primary care	Foreground	No
40	GP surgery diabetes review	Foreground	No
41	PWDI follow‐up in primary care	Foreground	No
42	Review discharge letter in primary care	Foreground	Yes
43	Request insulin/equipment prescription in primary care	Foreground	No
44	Supply insulin and equipment in primary care	Foreground	No
45	Prescribe insulin and equipment in primary care	Foreground	No
46	Seek assistance after discharge	Foreground	Yes
47	Adjust insulin following discharge	Foreground	Yes
48	Provide authority to administer insulin for district nurses	Foreground	No
49	Review referral in primary care	Foreground	No
50	Treat presenting illness	Foreground	No
51	Provide diabetes framework	Background	Yes
52	Empower PWDI management of diabetes	Background	Yes
53	Healthcare organisational capacity	Background	No
54	Manage workload	Background	No
55	Provide transfer of care infrastructure	Background	No
56	Maintain IT infrastructure	Background	Yes
57	Manage stock of insulin and equipment	Background	Yes
58	Provide appropriate competent staff	Background	Yes
59	Train staff around diabetes and insulin use	Background	Yes

### 3.1 Key targets for potential indicators

**FIGURE 3:**
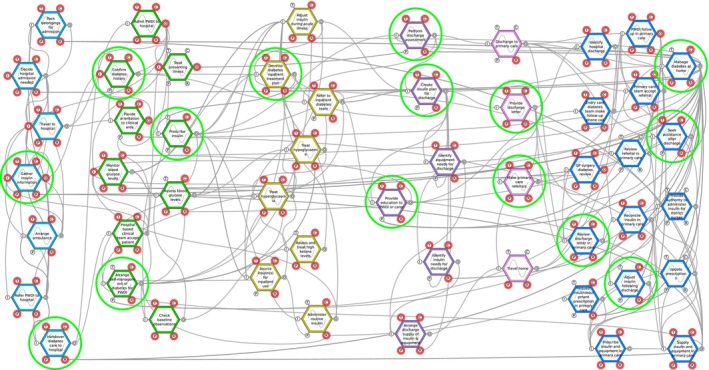
FRAM model of foreground functions. Targets for potential leading indicators are highlighted in yellow.

**FIGURE 4:**
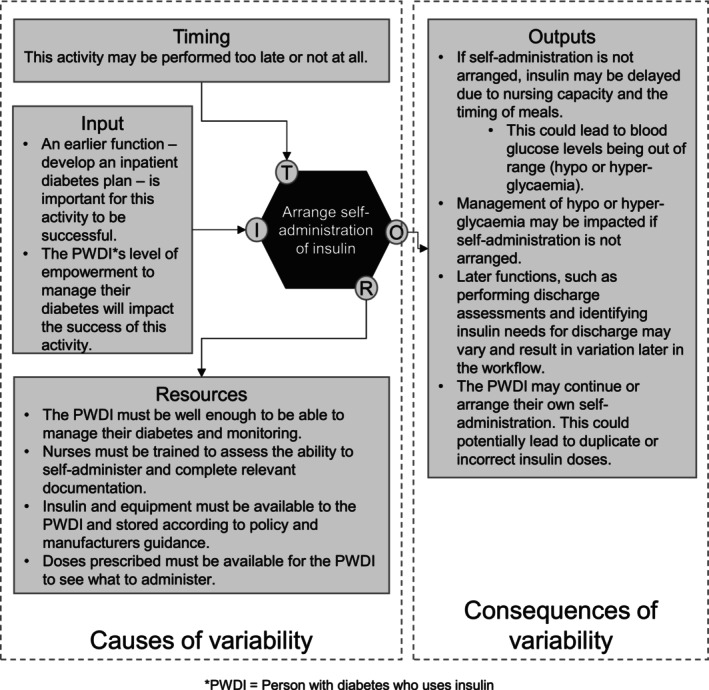
Potential causes and consequences of variation for the function ‘Arrange self‐administration of insulin (during hospital admission)’.

Six background functions and 15 foreground functions were associated with the greatest variability impacting insulin management across ToC. This variability was due to the potential for inaccuracy, incorrect timing or interactions with other functions. These functions are highlighted in Table 1. These were considered potential targets for developing leading indicators; see Table 2 for a list of these functions and their definitions. The FRAM model with targets for potential indicators is shown in Figure 3. An example of a function demonstrating the potential causes and consequences of variability is demonstrated in Figure 4.

**TABLE 2:** Functions considered as potential leading indicators and their definitions.

Type of function	Name of function	Definition
Foreground	Manage diabetes at home	Managing all aspects of diabetes care including: Collaborating to develop and update diabetes plan Monitoring glucose levels and identifying and treating hypoglycaemia Seeking advice if blood glucose levels are problematically outside of range (as per diabetes plan) Administering insulin and adjusting doses Maintaining sufficient insulin and equipment supplies Attending appointments for review Undertaking training to understand how to manage diabetes according to plan Storing insulin appropriately in fridge until cartridge/pen is in use
	Handover diabetes care to hospital	Communication of information: Includes the person with diabetes who uses insulin (PWDI)s current illness, medical and diabetes history and insulin information May be shared by the paramedics or by the general practitioner (GP) May be performed over the telephone, by email or by printed report
	Gather insulin information	Identify all relevant information about insulin that is available at the time depending on: The location of the PWDI The consciousness level of the PWDI Available resources (e.g., pen device and record book availability)
	Confirm diabetes history	Identify presence of diabetes: Identify past medical history and presence of diabetes Consider diabetes and glucose levels alongside signs and symptoms of illness Medication history and identify insulin use
	Develop diabetes inpatient treatment plan	Plan should describe an appropriate insulin regimen prescribed for current situation based on: Pre‐admission diabetes management Lifestyle factors Impact of current illness and concurrent medications reviewed Plan may include withholding insulin (for example if PWDI has hypoglycaemia), changing to intravenous insulin, or reducing the dose if unable to eat
	Prescribe insulin	Insulin is prescribed for inpatient administration along with rescue treatments using Electronic Health Record (EHR)
	Arrange self‐management of diabetes for PWDI while in hospital	Staff perform assessments, paperwork, and organisational requirements to enable PWDI to administer their own insulin. This includes: Assessing capacity and understanding Obtaining written consent Arranging suitable insulin and equipment to allow them to: ◦ Administer insulin doses ◦ Monitor blood glucose levels ◦ Manage hypo or hyperglycaemia
Foreground	Perform discharge assessment	Evaluate PWDIs insulin needs for discharge and consider: Whether any support is likely to be required given social circumstances and potential impact of illness on ability to manage insulin. Impact of illness and concomitant medications
	Create insulin plan for discharge	Develop plan with PWDI for managing diabetes after discharge considering: Insulin requirements during admission and blood glucose levels Diet in hospital and likely diet following discharge Other medications and their potential impact on insulin dosing Discharge assessment for social and other support needs Develop a plan that includes all the above plus: ◦ Details about which insulin(s) and device(s) to use ◦ What to do when unwell (sick day rules) ◦ Plan for who will administer insulin ◦ Monitoring requirements
	Provide discharge letter	Letter from hospital to GP including details of: Diabetes management during admission Changes to diabetes management and diabetes care plan for discharge List of medicines and insulin prescribed Other equipment not routinely prescribed at most hospitals (although some do) Discharge letters are written on electronic health records (EHR) and: Sent electronically to GP surgery email inbox A printed copy is given to the PWDI and/or caregiver
	Provide education to PWDI or carer	Provide education to PWDI or their caregiver including: How to monitor blood glucose levels How to administer insulin How to adjust insulin doses as needed What to do when unwell How to dispose of sharps Implications for driving
	Make primary care referrals	Referrals made to relevant outpatient teams where needed including: District nurses to help with insulin administration Community pharmacy for review of discharge medications
	Review discharge letter in primary care	Administrative staff in GP surgery: Identify hospital discharge letter Assign to task list of relevant clinical staff for review (e.g. clinician for review of diabetes, pharmacist, or Medicines Management Technician if medicines/insulin involved)
	Seek assistance after discharge	If an issue with diabetes or insulin occurs after discharge: PWDI, caregiver, GP or other healthcare professional seek help or advice to manage Advice could be sought from primary or secondary care
Foreground	Adjust insulin following discharge	Healthcare professional in collaboration with PWDI or caregiver: Review blood glucose levels and insulin doses Adjust insulin to ensure blood glucose levels stay within desired range as much as possible Update diabetes plan
Background	Provide diabetes framework	Provider strategies include organisational: Staffing policies including specialist teams Training provision Equipment and medication formulary Standard operating procedures and guidelines Commissioned pathways, their oversight and assurance
	Empower people who use insulin to manage their diabetes	Providing the training and support to enable PWDI (or their caregivers) to manage diabetes at home (see foreground function for included components)
	Maintain IT infrastructure	Provide and maintain a functional IT system and associated software and hardware that: Allows access to healthcare records across organisations Enables recording of and access to medical history, medications, appointment details, clinical letters, pathology, and laboratory results etc Includes the wireless connection between the monitoring devices and the hospital EHR system
	Manage stock of insulin and equipment	Ordering system in place within hospital or primary care pharmacies to: Ensure that insulin is ordered, stocked and stored appropriately Manage stock on wards Management and adjustment of guidelines where supply issues occur Insulin equipment is managed by: Community pharmacy when prescribed by GPs in primary care In hospital the manage provision of: ◦ Diabetes specialist nurses provide insulin equipment for the PWDI ◦ Hospital stock systems provide a supply of needles, syringes, sharps bins and monitoring devices etc
	Provide appropriate competent staff	Organisations provide adequate healthcare professionals with appropriate skills to match demand of patient population
	Train staff around diabetes and insulin use	Training for staff enables non‐specialist diabetes staff to be equipped with the competencies to care for PWDI using insulin

Abbreviations: PWDI, people with diabetes who use insulin.

Two representative functions were used as examples to consider possible measures at the Seminar and are presented below. These were ‘Arrange self‐administration for PWDI’, and ‘Empower PWDI to manage diabetes’.

### 3.2 Types of variability identified

Potential variability was identified in functions spanning the whole ToC care pathway. Very few functions were automated or had systems in place to support performance. Functions required many resources to be completed successfully. Empowered PWDI were key to providing safety information and could support staff by managing diabetes; however, other factors influenced how successfully their contributions were received. For example, the PWDI may be too unwell to contribute to their care during their acute illness, and hospital policies around the storage of medicines could interfere with enabling self‐administration. Alternatively, the formal function may not be completed, and self‐administration happens informally, but this can introduce variability through inaccurate documentation and potentially insufficient access to insulin, carbohydrates, and equipment needed. The availability of skilled staff with knowledge to understand insulin and diabetes management during acute illness was a key resource required for almost all functions. Policies and guidelines provide detailed guidance about many aspects of ToC; however, these were not always programmed into the EHR and required staff to know or access and act on the information within the guidelines. The consequences of unsuccessful functions across ToC included incorrect doses of insulin being administered, which led to hypo‐or hyperglycaemia. Unsuccessful functions also impacted later functions in the care pathway and therefore the successful management of insulin across ToC.

### 3.3 Seminar findings

Seminar subjects agreed that the six background functions and 15 foreground functions proposed were strong potential targets for developing indicators of safe insulin management across ToC. Subjects highlighted the challenges of safety measurement in general, including the many different pathways PWDI take to hospital admission and the fact that data is currently stored in multiple systems and formats. Central access to key information is not available to all who need it and changes in information governance arrangements will be required especially to ensure data can be viewed across the whole care pathways and that PWDI have appropriate access and ability to input relevant data. Minimal real‐time data is collected in the NHS and mechanisms to allow such collection will need to be developed. These digital systems would need both the PWDI and healthcare staff to see inclusion of data items such as glucose levels, insulin doses administered, diet and any treatments taken for hypo or hyperglycaemia across the care pathway to allow proactive intervention. Subjects identified there was a gap in measures reflecting “Empower PWDI to manage diabetes” (background function). Currently the blood test glycated haemoglobin (HbA1c) is used to understand diabetes management over time acting as a proxy for PWDI empowerment, but this is a retrospective measure as it represents average glucose control over previous months. It was agreed that proactive measures should be a target for development and that qualitative data would be essential to capture performance in these areas. Indicators that capture PWDI knowledge, belief and attitudes would be important and that indicators were person centric. A key challenge would be facilitating access for PWDI to shared systems such as EHR to allow recording of real‐time information around hypoglycaemia, diet and side effects which could be shared proactively with the healthcare team. Subjects identified the need for accurate recording of insulin self‐administration on hospital records to support measurement of the function “Arrange self‐management of diabetes while in hospital” (foreground function). No routine measures exist to capture the number of PWDI who self‐administer insulin and methods to collect these data require development. At present, PWDI have no way to enter relevant information themselves into the hospital EHR and rely on staff transcribing on their behalf.

For both functions, subjects felt that data on blood glucose levels and dosing information from continuous glucose monitors (CGM) and pen device recordings urgently need to be integrated into electronic patient records across the healthcare system. Findings from the seminar and the FRAM model were combined to produce a list of potential leading indicators for each of the two functions (Table 3).

**TABLE 3:** Example measures for Empowering People with diabetes who use insulin (PWDI) to self‐manage and arrange self‐management of diabetes in hospital.

Potential leading indicator	Activities involved	Potential measures	Target audience	Indicator type
Empowering PWDI to self‐manage	Collaborate to develop and update diabetes plans	Number of organisations with shared, co‐produced, documented diabetes plans in place	Integrated Care System	Passive
	PWDI attend appointments for review	Percentage of appointments scheduled. Percentage of appointments attended Timing of follow‐up after admission	Organisation PWDI Healthcare teams	Passive (Active for PWDI and healthcare teams)
	Undertake training to understand how to manage diabetes according to plan	Number of PWDI undertaking commissioned training Qualitative data from PWDI describing: ◦ Usefulness of training ◦ How well it met their needs ◦ Their understanding of diabetes management and motivation to manage diabetes and use insulin	Organisation and Integrated Care System	Passive
	Monitor glucose levels	Percentage of PWDI with diabetes regular prescriptions for monitoring equipment Population level glycated haemoglobin (HbA1c) data (percentages of population within different ranges)	Organisation	Passive
	Take insulin and adjust doses based on test results and other factors such as carbohydrate intake	Percentage of PWDI with a documented, up‐to‐date, co‐produced, shared diabetes plan	Organisation	Passive
	Treat hypoglycaemia and seek help or advice where blood glucose levels are problematically outside of range (as per diabetes plan)	Number of documented diabetes plans with directions for when to seek help or advice Number of contacts to diabetes teams or GP surgeries seeking advice for blood glucose levels	Organisation	Passive
	Maintain sufficient insulin and equipment supplies to continue to administer and monitor insulin	Insulin and equipment formularies agreed across region Regularly reviewed and updated Escalation plans and alternative options defined in case of supply shortages	Organisation	Passive
	Store insulin appropriately in fridge until cartridge/pen is in use	Evidence that insulin initiation guidelines and education programmes review and consider PWDIs ability to store insulin according to manufacturer directions	Organisation	Passive
	PWDI knowledge, belief and attitudes around diabetes and insulin management	Qualitative surveys and responses	Organisation Healthcare teams	Passive (Active for teams)
Arranging self‐management of diabetes in hospital	Recognition person uses insulin and should self‐administer unless there is a reason not to	PWDI and diabetes self‐management status highlighted on EHR ◦ Measure percentage of PWDI who are self‐managing their diabetes	Healthcare professional, teams and Organisation	Active (Passive for the organisation)
	Risk assessments performed to ensure self‐administration appropriate	The number of task(s) outstanding for completion highlighted on EHR	Healthcare professional and teams	Active
	Paperwork and other organisational requirements completed including: Assessment of insulin administration technique Arranging informed consent with PWDI Completing forms and documentation	The number of task(s) outstanding for completion highlighted on EHR	Healthcare professional and teams	Active
	Insulin and equipment provided to allow: Insulin administration Blood glucose monitoring Carbohydrates to treat hypoglycaemia	The number of task(s) outstanding for completion highlighted on EHR	Healthcare professional and teams	Active
	Identification and documentation of insulin doses taken on electronic health record	Number of doses documented on EHR Real‐time data for blood glucose levels Insulin doses or blood glucose levels outside normal range highlighted on EHR	PWDI, Healthcare professional and teams	Active
	Identification and documentation of any blood glucose levels outside desired range and any carbohydrates taken to treat hypoglycaemia	Real‐time data for blood glucose levels Insulin doses or blood glucose levels outside normal range highlighted on EHR Carbohydrates consumed by PWDI documented on EHR Carbohydrates consumed by PWDI to manage hypoglycaemia highlighted on EHR	PWDI, Healthcare professional and teams	Active
	PWDI highlighting any issues to nurses or doctors	Number of queries from PWDI about diabetes or insulin management	Healthcare professional and teams	Active
	Insulin doses adjusted in agreement with PWDI	Number of dose changes highlighted in EHR Number of PWDI signatures highlighting agreement for dose change Real‐time data on accuracy of dose documented compared with dose prescribed on EHR	PWDI, Healthcare professional and teams	Active

## 4 DISCUSSION

A new approach, the Functional Resilience Analysis Method (FRAM) was applied successfully to model insulin management across ToC. Challenges in managing insulin safety vary greatly during a PWDI's journey through admission and following discharge. By focusing on the full journey, the PWDI's experience was made central and was considered in its entirety. The process of developing the FRAM model and potential indicators allowed patients, healthcare professionals, and other stakeholders to share the real‐life clinical situations that impact safe insulin management and the issues that matter to them. Detailed descriptions of the challenges identified can be viewed in the Table S1. We found potential areas for developing proactive indicators to highlight risks in real time, providing key opportunities for safety improvement. Analysis of the FRAM model identified 15 highly variable foreground functions as potential targets for the development of active leading indicators and six background functions as targets for passive leading indicator development. These functions had the greatest impact on outcomes. Examples of potential measures for further development were identified. Safe insulin management across ToC relies on the inclusion and empowerment of PWDI and their caregivers.^12^ Therefore, co‐developing new leading indicators for safe insulin management with this community is essential.

We demonstrated how the FRAM model can be used in a collaborative way as a basis to work with PWDI, caregivers, and healthcare professionals to identify gaps in safety measurement, potential new measures, and means of data collection to identify challenges to overcome. These findings can be fed into the next stage of indicator development of defining the measures, ensuring their purpose is clear, what the units of measurement will be, how the data will be collected and calculated, and how such data will enable people involved to monitor and anticipate potential safety issues.^22^ The application of FRAM provided a method to identify potential indicators based on understanding how work is performed and how variability can impact outcomes later in the pathway. It contrasts with other approaches to indicator development that rely on analysis of past harm. Applying this method is challenging without the input of an experienced practitioner, and given the extensive variation identified across almost all functions, it was necessary to focus on representative functions or the model would become overwhelming. Those wishing to use this method would benefit from the development of training materials and mentorship models, which should support potential users to understand how and when to use this method to get the most benefit.

For safety improvement interventions to be effective, the causes of variability influencing successful outcomes must be understood. Leading indicators can highlight this variability, providing opportunities to intervene and evaluate improvement. Potential real‐time measurement is limited by the technology and integration of current systems. As EHR and wearable technologies such as CGM become more compatible and connected within and across care settings, the opportunities for active leading indicators and real‐time measures will expand. Insulin management is undergoing significant transformation with the advent of CGM. CGM allows glucose levels to be monitored through a device attached to the skin, and results are sent to an application automatically. Such devices are not currently routinely integrated into electronic health records (EHR) and are not universally used for all people with diabetes who use insulin; however, researchers are exploring the safety and potential benefits of this approach.^23^, ^24^, ^25^, ^26^, ^27^ As such technology becomes more widely used and more integrated across health care systems, the FRAM model developed in this process will require adaptation.

Using FRAM to develop leading indicators across ToC allows a proactive perspective of safety improvement that provides a strong foundation for indicator development. This method meets many of the Global Principles for Measuring Patient Safety^28^: It seeks to target key areas for improvement, the process requires full involvement of PWDI and their caregivers, it considers the whole journey across different care settings, and it aims to identify real‐time data. Further work to develop specific measures should strive to meet the other aims of ensuring equity and that they can be continuously adapted to changes in care pathways. In addition, the burden of data collection for staff must be minimised.

A FRAM model allows potential outcomes in a care pathway to be anticipated. It can demonstrate how functions promote successful outcomes (e.g., enabling self‐administration in hospital) and how others can cause adverse outcomes if omitted or delayed (create insulin plan). Several challenges limit the opportunities for FRAM to be used more widely within the NHS and other healthcare systems. The first is the limited training opportunities to learn how to use and apply FRAM. There are currently few (if any) courses available to learn how to develop a FRAM model in England. Guidance is based on written materials and/or ad hoc peer support from those who have already used the method. Given the lengthy process and multiple steps involved, opportunities for training and formal mentoring would support those who wish to use FRAM to develop the skills and knowledge to get the most out of the process. In England, the NHS has introduced the role of the Patient Safety Specialist,^29^ who may be a suitable target audience for such training. Another practical challenge is the resource implications for gathering and analysing data, then performing and validating the FRAM. Each of these steps requires input from stakeholders to ensure that findings represent how work is performed in real‐life settings. Given the financial, workforce, and workload pressures facing the NHS and other healthcare systems, the use of FRAM will need to be carefully targeted to care pathways that will obtain the most benefit. Finally, FRAM models may be large and difficult to interpret, and therefore presenting information meaningfully to influence change may be challenging.^30^, ^31^

### 4.1 Strengths and limitations

This study used multiple methods to model insulin management across ToC. PWDI and healthcare professionals across different care settings contributed to the development and validation of the model. Due to the high level of detail involved in a FRAM analysis, the model produced is specific to the study. As interview, focus group and seminar subjects were recruited from across England, the model produced is likely to apply to many integrated care systems in England. Although many of the principles will be similar in other healthcare settings, the detailed results may not be generalisable to other healthcare systems.

Due to the pressures on clinical staff, it was challenging to get engagement, and it was not possible to perform observation in primary care. Using the NRLS data to validate the FRAM model allowed primary care settings to be represented but identified the need to perform additional work with district nurses to fully map the functions that occur in this part of insulin management during ToC.

## 5 CONCLUSION

Due to the complexity of managing insulin across ToC, there are significant and persistent real‐life challenges for all involved. Without data to visualise where issues are occurring, it is difficult to understand the scope of these issues or make and evaluate improvements. This study successfully applied FRAM to identify potential areas to target active and passive leading indicators for safe insulin management during TOC. The method provided valuable insight into how and where variability occurs, and how safety is maintained despite variability, but was lengthy and specific to the context in which it was developed. Example potential measures were described; however, a coproduction approach to expanding, defining, and validating these is required.

## FUNDING INFORMATION

This report is independent research funded by the National Institute for Health and Care Research ARC North Thames.

## CONFLICT OF INTEREST STATEMENT

The authors declare no conflicts of interest.

## Supporting information


**Table S1.** Sample of functions and variability.

**Data S1.** Data sources.

**Data S2.** Incident data.
