# Supplementary material for: A new approach to identifying safety measures across transfers of care for people who use insulin for Type 2 diabetes
Source: Diabet Med. 2025 Jul 22;42(10):e70101. doi: 10.1111/dme.70101 (PMC12434425; doi:10.1111/dme.70101)
Supplement: Supplementary file 1 — Table S1. Sample of functions and variability. [file DME-42-e70101-s002.docx]

### Supplementary Table 1: Sample of functions and variability

| **Function** | **Causes of variability** | **Impact of or on other functions** | **Resources required** | **What is controlling the function?** |
| --- | --- | --- | --- | --- |
| Confirm diabetes history | The ability to confirm that someone has diabetes and how they manage this relies on the PWDI or their caregiver being able to provide this information. Some details are available in the GP record, for example the type of insulin and the device used, sometimes dosing information may be included. Information about how insulin is adjusted based on diet and exercise is known only to those managing this, usually the PWDI or their caregiver.  Insulin dosing information for people who have insulin administered by district nurses is held in the district nursing records which may not be available to the hospital staff.  If staff are unaware or do not identify that someone has diabetes and requires insulin, there may be delays to prescribing and administration of doses, and consequently unstable blood glucose levels.  If staff are unable to identify previous doses of insulin, the PWDI may be administered an intravenous insulin infusion. | This may impact on the appropriateness of #13 Diabetes inpatient treatment plan developed. It may lead to omission of #14 Prescribe insulin and #16 Assess blood glucose levels, and consequently on #17 Treat hypoglycaemia & #18 Treat hyperglycaemia. If diabetes history is not confirmed, it may impact the ability to # 19 Arrange self-administration of insulin. | Staff available to seek information and discuss medical and diabetes history with PWDI or their caregiver,  Electronic health records (EHR) containing details of diagnosis of diabetes (including type and prescribed treatments)  IT equipment, such as functioning computer stations, smartcard readers (allowing access to EHR), keyboards available  Staff available must have the skills to identify the tasks required, for example confirming diabetes history, and prioritise these according to the needs of all the patients on their caseload. | Staff diabetes knowledge is essential to understand the need to identify the PWDI usual diabetes management and consider how their current circumstances require adjustments.  Diabetes policy and guidelines provide information to staff about how to manage insulin and diabetes in different situations. |
| Arrange self-administration for PWDI | Self-administration of insulin may not be arranged. PWDI insulin may be locked away or stored in the fridge in the clinical area where they are unable to access it to administer doses at the time needed. The PWDI also requires access to equipment to check blood glucose levels and treat hypoglycaemia. Manufacturers require insulin cartridges and pre-filled pens to be stored in the fridge until they are used, after which they can be kept at room temperature for 28 days.  To arrange self-administration, nursing staff must feel confident that the PWDI is able to manage the tasks involved in the context of their skills and knowledge, but particularly in the context of their often fluctuating health, where illness may temporarily prevent their ability to manage.  Where there are delays in providing and administering insulin in hospital, PWDI may arrange and source their own supply of insulin and administer doses to themselves. There is the potential risk of duplicate doses being administered, or EHR records not being accurate if doses administered by PWDI are not recorded. | This function requires #13 Diabetes inpatient treatment plan developed to have been completed. Where PWDI can and are authorised to self-administer, this can impact the following functions:  #24 Administer routine insulin.  #23 Adjust insulin during acute illness.  #17 Treat hypoglycaemia.  #18 Treat hyperglycaemia.  #25 Perform discharge assessment.  #26 Identify insulin needs for discharge.  #27 Create insulin plan for discharge. | The correct insulin and a suitable administration device must be available for the PWDI to use, and must be in date.  Equipment available to administer insulin, monitor blood glucose levels and dispose of sharps are required.  A lockable patient medicine cabinet that can be accessed by the PWDI is required to allow organisations to meet legislative and hospital requirements for safe storage of medications.  Staff must be available to complete the required assessments, documentation and activities involved in sourcing insulin, assessing ability and capacity to self-manage, and transcribe and document doses administered.  The specialist diabetes team available are required to provide expert advice where ward staff are unsure or are facing a complex situation. | Diabetes policy and guidelines provide information to staff about how to manage insulin and diabetes in different situations.  Provider organisation diabetes strategies will provide guidelines and authority for how to assess and authorise a PWDI to self-manage their insulin and diabetes.  Legislation and relevant organisational policies impacts how insulin should be stored and how PWDI can have access to it in order to self-manage their diabetes.  Diabetes training for PWDI and staff enables understanding of how diabetes should be managed, and how doses should be adjusted based on varying glucose levels.  The local insulin and equipment formulary defines what insulins and equipment are available for PWDI to use while in hospital. |
| Provide education to PWDI or carer | Variation in providing education to the PWDI or caregiver can occur where the need for education is not identified, for example someone who has previously been using insulin, but either had a pre-existing need for additional information, or where changes to insulin management have occurred during the hospital admission and the need to provide education around these was not recognised.  The provision of education is dependent on timing and the availability of trained staff. The Specialist Diabetes Team did not have capacity to provide training outside core working ours, for example over night and sometimes over weekends. Where team members were unavailable due to industrial action or illness, remaining staff members had to prioritise education for those newly started on insulin or where a specific referral had been made.  The specialist diabetes team had a structured method of providing education that ward staff did not have access to, therefore the depth and comprehensiveness of training varied depending on who was providing the information.  Some PWDI may require additional education sessions due to the large amount of information shared, the ability to provide this relied on staff recognising the additional need, the timing of discharge and the availability of staff able to provide the education. | Providing education to the PWDI or carer is an opportunity to confirm that the correct insulin and devices have been provided. Therefore, variability in this function can impact:  #26 Identify insulin needs for discharge.  #28 Identify equipment needs for discharge.  #36 Self-management of insulin in primary care  #8 Monitor blood sugar levels.  #16 Assess blood glucose levels.  #17 Treat hypoglycaemia.  #18 Treat hyperglycaemia.  #24 Administer routine insulin | To provide education to the PWDI and/or their carer, this requires staff trained to identify the need for such education, and to provide the training. Training around insulin requires teaching about monitoring blood glucose levels, understanding the results and how to manage insulin based on blood glucose levels. PWDI must be trained to understand the signs and symptoms of hypo and hyperglycaemia and how to manage these conditions. It is important to explain how to use insulin when unwell, and when to seek extra help. The PWDI must also be able to use their insulin device to deliver the correct dose and dispose of the sharps correctly. Due to the bredth of the information and education required, the diabetes specialist teams are usually the team who provide this education.  Leaflets may be used to support the education, usually provided by manufacturers. Placebos and equipment are available to support the clinical staff to demonstrate techniques and provide education.  Electronic health records allow healthcare professionals to document details of training that has been provided.  Telephones are required to request training and are used to contact the PWDI after discharge to follow-up if required. Insulin passports are available to allow PWDI to document their insulin details, however these were not observed in use during this study. | Diabetes policy and guidelines Formularies Diabetes training Commissioning contracts (e.g. 14 day supply in contract with commissioners) |
| Train staff around diabetes and insulin use | Staff training around diabetes and insulin use is provided during undergraduate education, by specialist diabetes teams and through an electronic learning module. Despite this training, many staff remained under-confident in managing and adjusting insulin. The diabetes specialist team were often consulted for advice on how to manage and adjust insulin for PWDI during their admission.  Undergraduate training could vary in terms of content, and staff may have graduated ad different times, meaning that it was variably recent and memorable. Staff have access to a nationally available e-learning package, but different organisations mandate this in different ways, leading to variability in completion. Training provided by the diabetes specialist teams is managed by that team and varies by organisation.  Evaluation of the impact of training requires assessment of competency. | The availability of staff who were trained, competent and confident in managing insulin and diabetes impacted most functions in the FRAM model. | The content of training is informed by national and local policies and guidelines.  Provision of training requires staff time to complete, and where provide by the local diabetes team, time to prepare and deliver the sessions. | The provision of training is controlled by the organisational priority setting, and their local diabetes strategy. National training and competency standards govern undergraduate training. |
