# Supplementary material for: A new approach to identifying safety measures across transfers of care for people who use insulin for Type 2 diabetes
Source: Diabet Med. 2025 Jul 22;42(10):e70101. doi: 10.1111/dme.70101 (PMC12434425; doi:10.1111/dme.70101)
Supplement: Supplementary file 3 — Data S2. Incident data. [file DME-42-e70101-s003.docx]

### Supplementary information 2: Incident data

The following incidents were selected from the NRLS data to represent different aspects of PWDI journeys across ToC.

1. A PWDI was admitted to hospital with gastroenteritis. Because he was eating and drinking less, his insulin doses were reduced. He was discharged home without being referred to the district nurses. He was already on the district nursing list of patients from before admission. When a district nurse visited him, the difference between the hospital doses and their records was identified. The GP was requested to update their prescription and the district nurse monitored the PWDI’s blood sugars. The GP reviewed blood sugars and discharge letter and decided that the PWDI should return to his previous insulin dose now that the gastroenteritis had resolved.
2. A person was started on insulin during a hospital admission, but no referral was made to the district nurses. The PWDIs daughter was not taught how to manage diabetes and insulin. The GP made a referral to the district nurses to provide training for diabetes management and to administer insulin.
3. A PWDI was admitted to hospital for surgery. Before the surgery she was fasting. The nurse administered her normal dose of insulin, despite the PWDI asking whether she should have this dose. The lady subsequently experienced hypoglycaemia and required an infusion of glucose. A referral was made to the hospital diabetes specialist nurses who advised on further management.
4. A PWDI was admitted to hospital. His usual insulin doses were prescribed to be given only “when required.” He missed several doses of insulin before this was identified.
5. A PWDI was due to have surgery. Before he went into hospital, he was unable to source his normal insulin and missed about two doses. When he was admitted to hospital, the ward did not stock his normal insulin, and he missed further doses. He was not eating or drinking because he was due to have surgery, and when his blood sugars were checked, they were very high. This was treated with an insulin infusion. His normal insulins were supplied by the pharmacy, but there was a delay in administering them.
6. A PWDI attended the emergency department. His family were with him. When he was moved to the ward, a nurse tried to identify whether he was due to have his insulin dose. His family said that he had been given his insulin, but this had not been documented on the electronic health record.
7. Insulins and other medications were given to a PWDI ready to be discharged from the hospital. While waiting to go, his blood glucose levels were checked and were high. The PWDI took his insulins to manage the high levels. He was kept in hospital for three hours to monitor his blood glucose.
8. A PWDI started on insulin was discharged from hospital. He noticed that his blood glucose levels were high despite administering his insulin. He went to the GP who identified that he had been given a placebo insulin pen that had been used for training to take home, and was therefore not getting any insulin.
9. A PWDI was admitted to hospital. He usually took a mixed insulin (Mix 50), however because this was not kept on the ward, a doctor prescribed a smaller dose of the same type of mixed insulin but with different proportions (Mix 25). His blood glucose levels were monitored regularly to review the impact of this change. The pharmacists identified the change and arranged a supply of his normal insulin the following morning.
10. A PWDI was discharged from hospital without a supply of any equipment, needles, sharps bin, lancets or monitoring strips. The PWDI was then unable to monitor his blood glucose levels or administer his insulin.
